# Understanding the gut‐brain axis to mitigate cognitive frailty

**DOI:** 10.1002/alz.093563

**Published:** 2025-01-09

**Authors:** Abu Bakar Abdul Majeed, Kalavathy Ramasamy, Maw Pin Tan, Siong Meng LIm, Khor Min Hui, Ai Huey Tan, Nurul Izzati Ahmad Fadzuli

**Affiliations:** ^1^ Universiti Teknologi MARA (UiTM), Puncak Alam Malaysia; ^2^ University of Malaya, Kuala Lumpur Malaysia; ^3^ Universiti Teknologi MARA, Puncak Alam Malaysia

## Abstract

**Background:**

Gut microbiota modulation of the brain function may present an opportunity to devise preventive or treatment strategies to manage impairments such as cognitive frailty (CF). This study aims to uncover the relationship between CF, gut microbiota, intestinal permeability and proteome.

**Method:**

A total of 137 fecal samples of the elderly were collected, and subjected to DNA analysis, and enzyme‐linked immunosorbent assays (ELISA). Plasma samples were subjected to mass spectrometry proteomic analysis. The parameters of the subjects measured include functional reach test (FRT), handgrip strength (HGS), Visual Cognitive Assessment Test (VCAT), Montreal Cognitive Assessment (MoCA), timed up and go (TUG) and UCLA three‐item loneliness scale (UCLA‐3).

**Result:**

At the genus level, Alistipes which are potential drivers of dysbiosis, are significantly increased in CF subjects. Proteobacteria are also negatively linked to FRT, HGS, VCAT, and MoCA, but positively correlated to TUG and UCLA‐3. Lactoferrin was upregulated in pre‐frail subjects. The plasma apolipoprotein AI (Apo‐AI) was upregulated 5 times in the CF subjects.

**Conclusion:**

These findings provide evidence for dietary intervention to alter gut microbiota that may modulate cognitive status.

